# Influence of increasing SnO_2_ content on the mechanical, optical, and gamma-ray shielding characteristics of a lithium zinc borate glass system

**DOI:** 10.1038/s41598-022-05894-5

**Published:** 2022-02-02

**Authors:** M. I. Sayyed, Aljawhara H. Almuqrin, K. A. Mahmoud, A. S. Abouhaswa

**Affiliations:** 1 Department of Physics, Faculty of Science, Isra University, Amman, 11622 Jordan; 2 Department of Nuclear Medicine Research, Institute for Research and Medical Consultations (IRMC), Imam Abdulrahman Bin Faisal University, P.O. Box 1982, Dammam, 31441 Saudi Arabia; 3 Department of Physics, College of Science, Princess Nourah bint Abdulrahman University, P.O. Box 84428, Riyadh, 11671 Saudi Arabia; 4 Ural Federal University, Mira St., 19, Yekaterinburg, Russia 62002; 5 Nuclear Materials Authority, El-Maadi, P.O. Box 530, Cairo, Egypt; 6 Physics Department, Faculty of Science, Menoufia University, Shebin El-Koom, 32511 Menoufia Egypt

**Keywords:** Materials science, Physics

## Abstract

A series of six samples were prepared based on the chemical composition of 65B_2_O_3_ + 20ZnO + (15-x)LiF + xSnO_2 (_where x = 0, 0.25, 0.5, 0.75, 1, and 1.25 mol%) to study the role of SnO_2_ on enhancing the optical and radiation attenuation capacity of the prepared glasses. The preparation of the glass series was performed using the melt quenching method at 1100 °C for 60 min. The density of the fabricated samples was measured using an MH-300A densimeter. The optical parameters of the fabricated glasses were calculated based on the spectrum recorded by a Cary 5000 UV–Vis–NIR double beam spectrophotometer in a wavelength range of 200 to 3000 nm. Furthermore, Monte Carlo simulation code and the XCOM online database were used to estimate the gamma-ray shielding capacity of the fabricated samples from 0.244 to 2.506 MeV. The results show enhanced gamma-ray shielding capacity due to the replacement of LiF by SnO_2_. The linear attenuation coefficient at 0.244 MeV was enhanced from 0.352 to 0.389 cm^−1^. The half-value thickness of the investigated glasses decreased from 1.967 to 1.784 cm when the increasing addition of SnO_2_ from 0 to 1.25 mol%.

## Introduction

It is well understood that adding B_2_O_3_ to glass modifiers easily results in the formation of glass, and the resulting glass has several distinct physical and chemical properties, such as allowing visible light to pass through the glass and thus the possibility of using this type of glass in various optical applications. Borate-based glasses have notable characteristics, such as their high thermal stability, high chemical durability, low melting temperature, good mechanical stability and ease of fabrication. Furthermore, some heavy metal oxides are utilized to increase the chemical durability of these glasses and to lower their high phonon energy^1,2^. When ZnO is added to borate glasses, it plays dual roles, namely, a network former and network modifier; thus, ZnO enhances the physical characteristics of the host glass system, such as its thermal stability, and reduces the melting temperatures of the batches during fabrication.

Additionally, SnO_2_ is a technologically important material because it has a wide range of applications, including in photocatalytic systems, gas sensors, and photovoltaic cells. Moreover, when SnO_2_ is added to B_2_O_3_, a comparatively high density is observed, which promotes its use as an efficient shielding material. From the nineteenth century until the present, various radioactive isotopes emitting ionizing radiation have been widely employed in nuclear medicine, dentistry, agriculture, industry, sterilization, food, and other disciplines that are difficult to list^3–5^.

Borate-based glass systems are largely studied in the literature since they have a wide range of applications in different fields, such as medical treatment and research, industrial technology, and engineering. These applications, especially the medical applications, use ionizing radiation. Workers in these areas are exposed to different types of radiation at different doses and varying energies; thus, they may experience health problems, such as cancers^6^.

As a result, there is an urgent need to deploy protective radiation shields to safeguard nuclear power plant workers, medical professionals, and patients in radiotherapy facilities from the dangers of radiation^7–10^.

Concrete and lead have historically been the most extensively utilized barriers for radiation protection since they have high photon attenuation ability. As a result, they are widely utilized as a protective barrier for gamma radiation and X-rays in a variety of forms, such as plates, tubes and bricks. However, concrete and lead are opaque to visible light; thus, they prevent light from passing through^11–13^.

To overcome the opaqueness problem of these two materials, researchers have resorted to developing new types of radiation-protective materials using different types of glass because glass has the ability to transmit light^14–16^. Recently, several research groups have developed multiple glass systems and studied the ability of these glass systems to attenuate radiation and thus reduce its harmful effects on humans and the environment^17–19^.

When studying radiation attenuation qualities, researchers identify fundamental quantities, such as the mass attenuation coefficient and some associated numbers. This amount may be calculated in a variety of ways, the most significant of which is a practical approach that involves exposing the sample to photons. A detector can be used to determine the number of photons that have penetrated the sample.

The Monte Carlo simulation approach is another option^20^. This approach involves creating a virtual reality for the practical experiment taking place in the laboratory. This approach differs from the practical way in that it saves time and effort while also lowering the likelihood of individuals becoming exposed to photons. Its significance is heightened by the shortage of equipment and facilities, forcing more researchers to rely on simulation. In the last few years, several researchers have adopted Monte Carlo simulations to investigate the radiation attenuation performance of borate glasses with different additives. For example, Mahmoud et al.^21^ used MCNP5 to examine the influence of CdO on the attenuation shielding performance of alkali borate glasses. They found that the addition of more CdO enhanced the radiation attenuation performance of the selected glasses. Acikgoz et al.^22^ prepared new alumina borate glasses, and when using MCNP5, they determined the role of CeO_2_ and Er_2_O_3_ on the radiation shielding performance of the prepared glasses. The authors concluded that glasses that contained Er_2_O_3_ had a higher mass attenuation coefficient than glasses with CeO_2_. Rammah et al.^23^ studied the impact of Bi_2_O_3_ and PbO on the radiation attenuation parameters of borate glasses. They used Monte Carlo simulation to investigate the attenuation factors at different energies ranging from 15 keV up to 15 MeV. The authors reported that the addition of PbO to borate glass enhanced the protective features of the samples to nuclear radiation. Stalin et al.^24^ prepared new lithium-borate glasses using the quenching method and simulated the radiation attenuation factors via the MCNP5 code. The authors examined the influence of replacing Bi_2_O_3_ with WO_3_ on the half-value layer and other related parameters. According to their results, the glass that contained 60 mol% B_2_O_3_ and 20 mol% Li_2_O and Bi_2_O_3_ had the smallest half-value layer. Asadi and Hosseini^25^ used the MCNP code to simulate the radiation attenuation coefficients of borate glasses doped with ZnO, Li_2_O and Bi_2_O_3_ in the energy range of 200 keV to 1.5 MeV. According to their results, glass with a composition of 20ZnO-50Bi_2_O_3_-15Li_2_O-15B_2_O_3_ had the shortest mean free path. Abou Hussein et al.^26^ simulated the radiation attenuation coefficients of borate glasses doped with one of the following oxides: V_2_O_5_, Cr_2_O_3_, Fe_2_O_3_ and TiO_2_. The authors concluded that a 3 cm-thick glass sample containing Cr_2_O_3_ was the best attenuator and could reduce the lifetime risk to cancer by five times.

In continuation of the previous efforts made by researchers to study the radioactive attenuation properties of borate glasses using Monte Carlo simulation, enrich the scientific community with more studies on the radiation attenuation properties of this kind of glass, and check the possibility of developing new shielding glasses based on borate glass, the Monte Carlo simulation code is utilized to estimate the gamma-ray shielding capacity of the novel lithium-zinc borate glass system. Moreover, to understand the optical properties of the developed glasses, the optical absorption band was measured in the wavelength range of 200 to 3000 nm. Additionally, the Makishima-Makenzie model was applied to predict the elastic moduli and microhardness of the fabricated glasses.

## Experimental techniques

### Glass fabrication, characterization, and optical properties

Tin-doped lithium zinc borate glass samples with nominal compositions of 65B_2_O_3_ + 20ZnO + (15-x)LiF + xSnO_2_ (where x = 0, 0.25, 0.5, 0.75, 1 and 1.25 mol%) were prepared. The glass composition and chemicals used to prepare samples along with glass code are shown in Table 1. Approximately 20 g of the batch composition of these chemicals was mixed well in an agate mortar for 60 min and then preheated at 350 °C for 60 min. This homogenous mixture was then heated in a porcelain crucible at 1100 °C for 30 min before being cast onto a stainless steel mold to generate glass discs. After quenching, the samples were immediately transported to a muffle furnace set at 300 °C for annealing.

**Table 1:** Chemical composition and density of the prepared BZLSn glasses.

Sample	Chemical composition mol%				Density (gm/cm^3^)	Molecular weight (gm/mol)	Molar volume (cm^3^/mol)
	B_2_O_3_	ZnO	LiF	SnO_2_			
BZLSn0	65	20	15	0	3.025	65.426	21.628
BZLSn1	65	20	14.75	0.25	3.052	65.738	21.539
BZLSn2	65	20	14.5	0.5	3.083	66.049	21.424
BZLSn3	65	20	14.25	0.75	3.131	66.362	21.195
BZLSn4	65	20	14	1	3.199	66.674	20.842
BZLSn5	65	20	13.75	1.25	3.265	66.986	20.516

The densities of the glasses were measured using the MH-300A densimeter according to Archimedes' rule, as shown in Eq. (1). Toluene was used at room temperature as an immersion liquid during the density measurements.

1 $$\rho_{glass} = \frac{{W_{a} }}{{W_{a} - W_{b} }}\rho_{tolune}$$

where W_a_ and W_b_ are the weights of the glass sample in air and in liquid toluene, respectively. The measured density, as well as the calculated molecular weight and molar volume, are listed in Table 1 for the fabricated BZLSn glasses.

The optical absorption data of the glass samples were obtained for the polished glass samples using a Cary 5000 UV–Vis–NIR double beam spectrophotometer.

### Gamma-ray shielding capacity evaluations

Both Monte Carlo simulation and the XCOM theoretical program were used to predict and affirm the photon shielding capacity of current BZLSn glasses. The calculation using the XCOM program depended only on the chemical composition of the fabricated glass system. After that, the calculation for the attenuation coefficient depended on the mixture, as presented in Eq. (2).

2 $$\mu_{m} \left( {\frac{{cm^{2} }}{g}} \right) = \mathop \sum \limits_{i} \omega_{i} \times (\mu_{m} )_{i}$$

where $\omega_{i}$ is the fractional abundance of the ith element in the shielding material.

**Figure 1:**
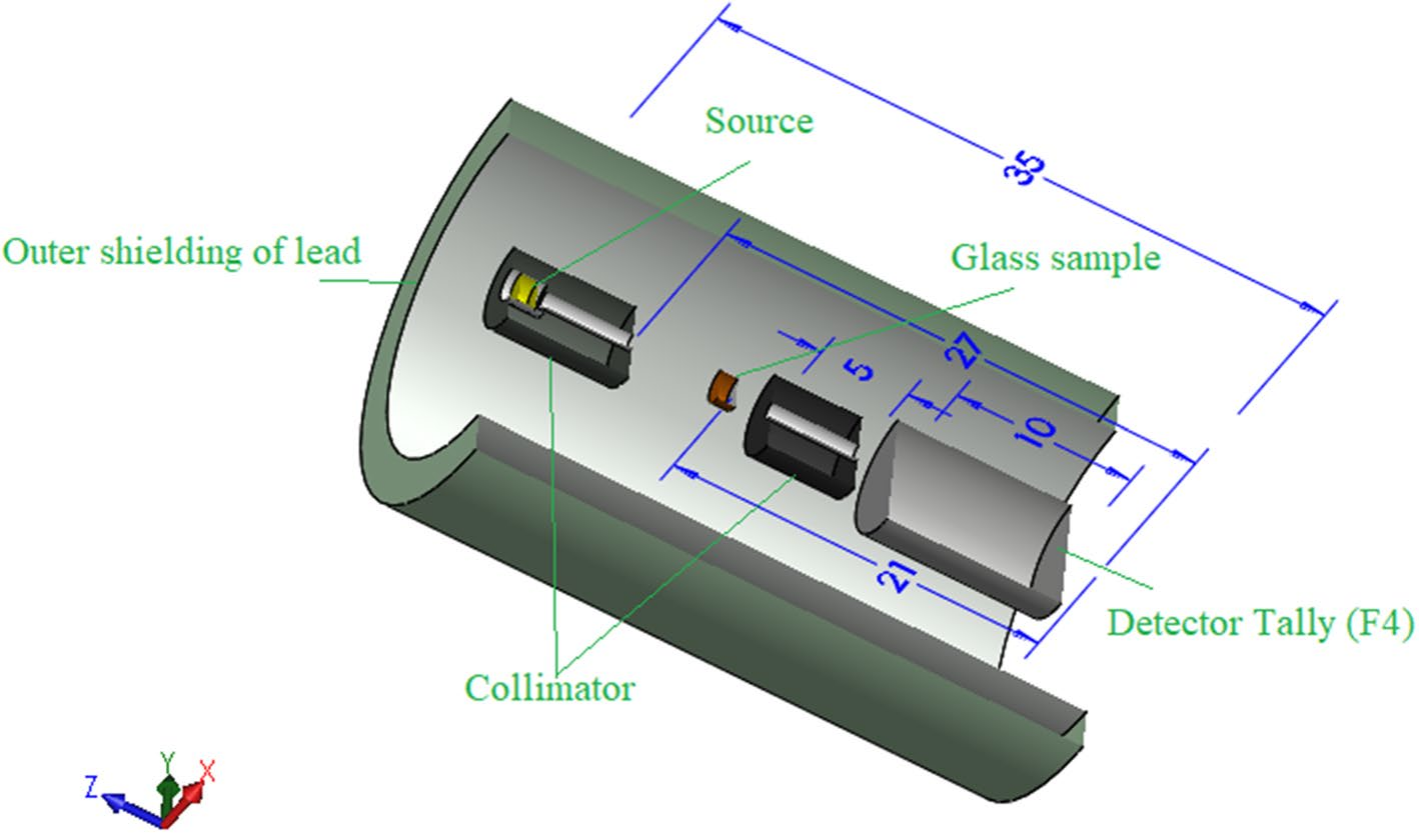
3D geometry representing the Monte Carlo simulation input file.

On the other hand, the Monte Carlo simulation code is based on many parameters, such as the sample composition, sample density, problem geometry, and ENDF/B-VIII library, from which the cross-section of interaction is extracted for various elements. The Monte Carlo simulation geometry consists of many cards that describe all components required for the simulation process, as illustrated in Fig. 1. The first important card is the cell card, which introduces the cell type, density, and boundaries. The mentioned figure shows many cells inside the prepared geometry, such as the collimators of lead with a density of 11.34 g/cm^3^, glass samples with their densities listed in Table 1, a radioactive source, and F4 detector tally to estimate the photons average track length per unit cell of the detector. The second is the surface card, which usually describes the shape and dimensions of the cells introduced in the first step. The dimensions of the various geometric components are illustrated in the figure. The third is the material card that introduces chemical composition of each cell in the arranged geometry. The source card also describes the source position, type, energy, decay probability, and emission distribution. The cutoff card is one of the physical cards used in the prepared geometry, and it is set to stop interaction after 106 historical cards.

## Results and discussion

### Physical and optical properties

**Figure 2:**
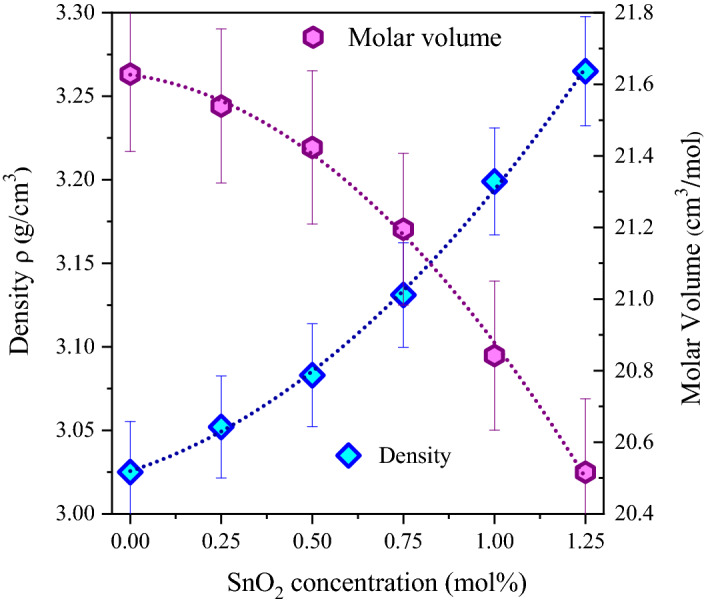
Density and molar volume of the glass system with various SnO_2_ concentrations.

The density and molar volume of the 65B_2_O_3_ + 20ZnO + (15-x) LiF + xSnO_2_ glass system with various SnO_2_ concentrations are shown in Fig. 2. The density of the sample improves as the SnO_2_ content (x) is increased; on the other hand, the molar volume decreases. The density of the fabricated glasses slightly increase from 3.025 ± 0.151 to 3.265 + 0.163 g/cm^3^, increasing the SnO_2_ insertion ratio from 0 to 1.25 mol%. The substitution of Li^2+^ ions (ρ = 0.534 g/cm^3^) with Sn^2+^ ions (ρ = 7.31 g/cm^3^) causes a slight increase in the density of the fabricated glass. Additionally, the increase in the fabricated glass density may be related to a significant decrease in the interatomic distance due to the substitution of LiF by TeO_2_.

**Figure 3:**
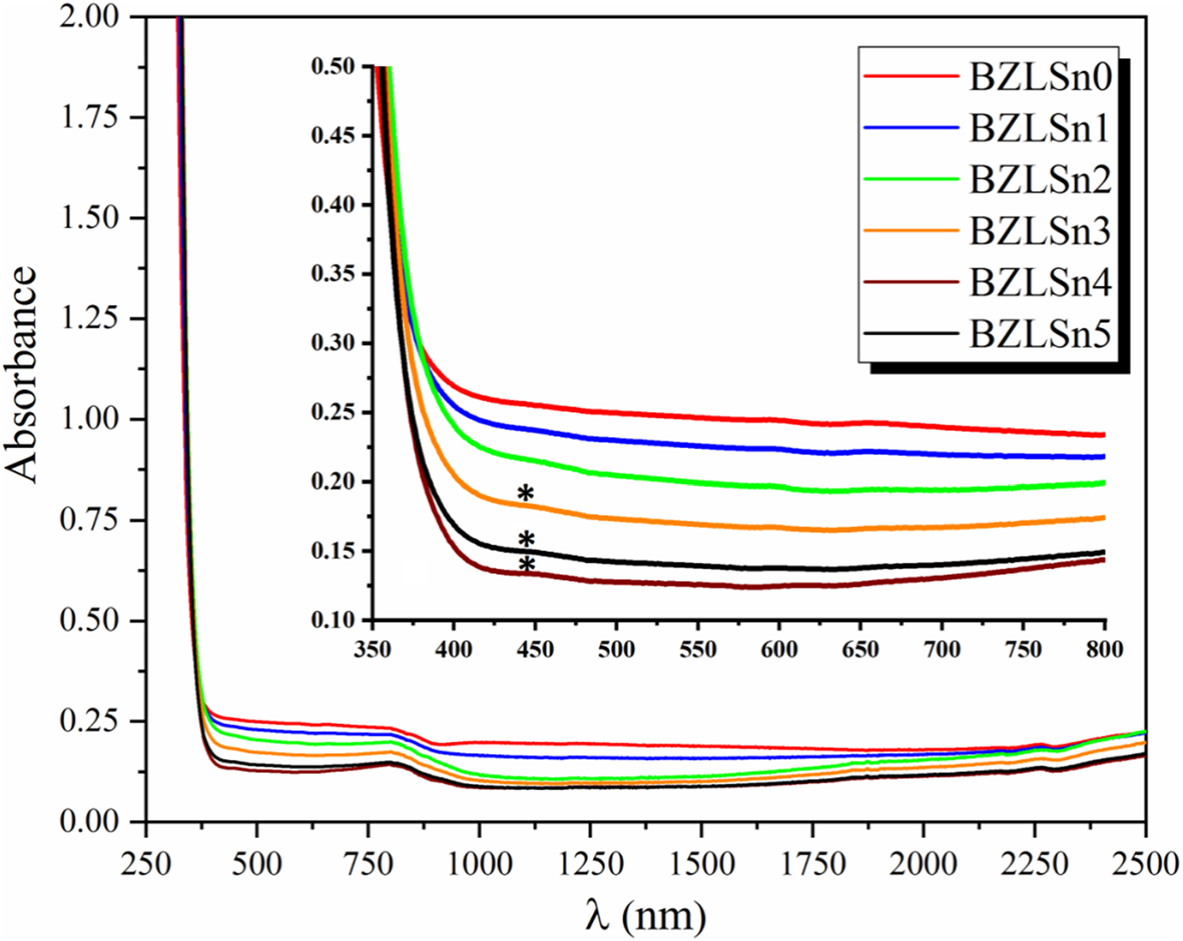
Optical absorption spectra of the BZLSn samples (inset: absorption between 350 and 800 nm).

Figure 3 shows the UV–Vis absorption spectra of the produced SnO_2_-doped lithium zinc borate glass samples, and the inset in Fig. 3 shows the absorption spectra from 350 to 800 nm. These measurements were carried out on samples with a thickness of 2.2–2.5 mm. The spectrum shows that the produced samples have significant UV absorption and that the strength of the absorption decreases as the concentration of SnO_2_ is increased^27^. As the SnO_2_ substitution ratio is increased, distinctive bands of SnO_2_ are observable in these glass samples: the peak at 445 nm (*) is attributable to a ligand in the metal charge assigning transition (Sn^4+^).

Tauc's rule, which Mott and Davis^28,29^ have modified, is shown in Eq. (3). This rule is used to estimate the optical energy band gaps for the investigated SnO_2_-doped lithium zinc borate glass samples in this study:

3 $$\left( {\alpha \left( \nu \right)h\nu } \right)^{m} = C\left( {h\nu - E_{g} } \right)$$

where m represents the kind of electronic transition. In this case, m = 2 indicates a direct allowable transition and m = 0.5 indicates an indirect allowable transition.

**Figure 4:**
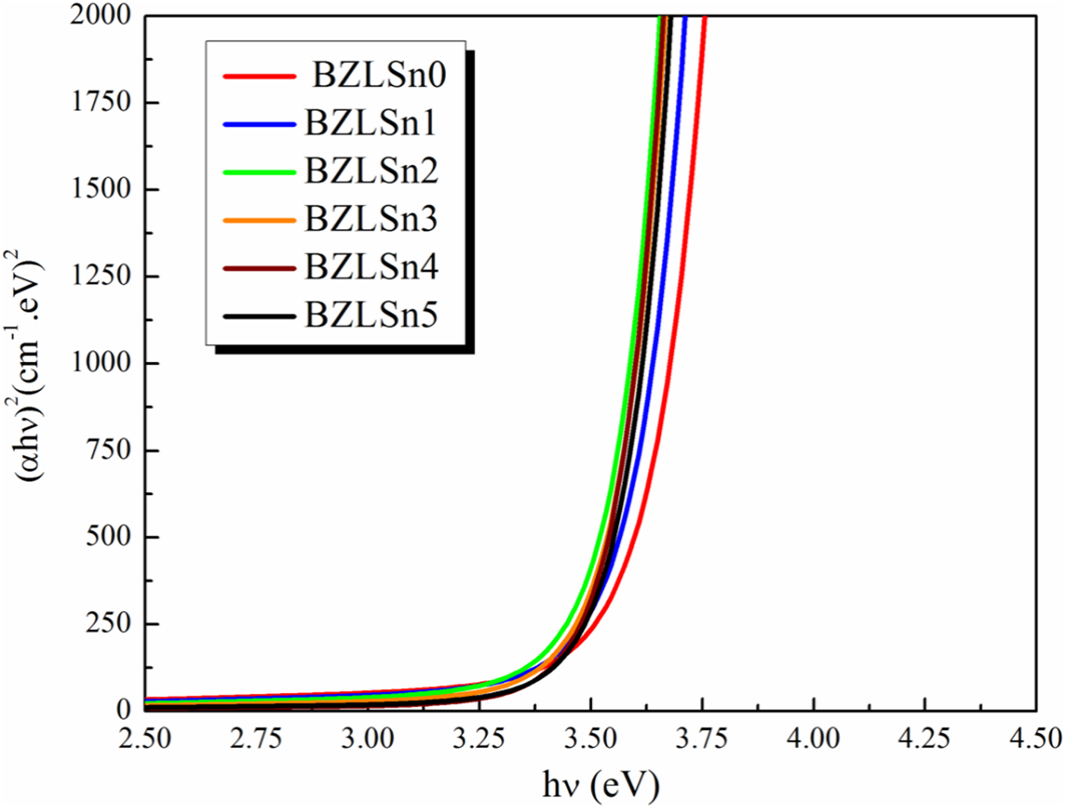
Dependence of (αhν) on (hν) for the direct allowed transition of the examined glass samples.

**Figure 5:**
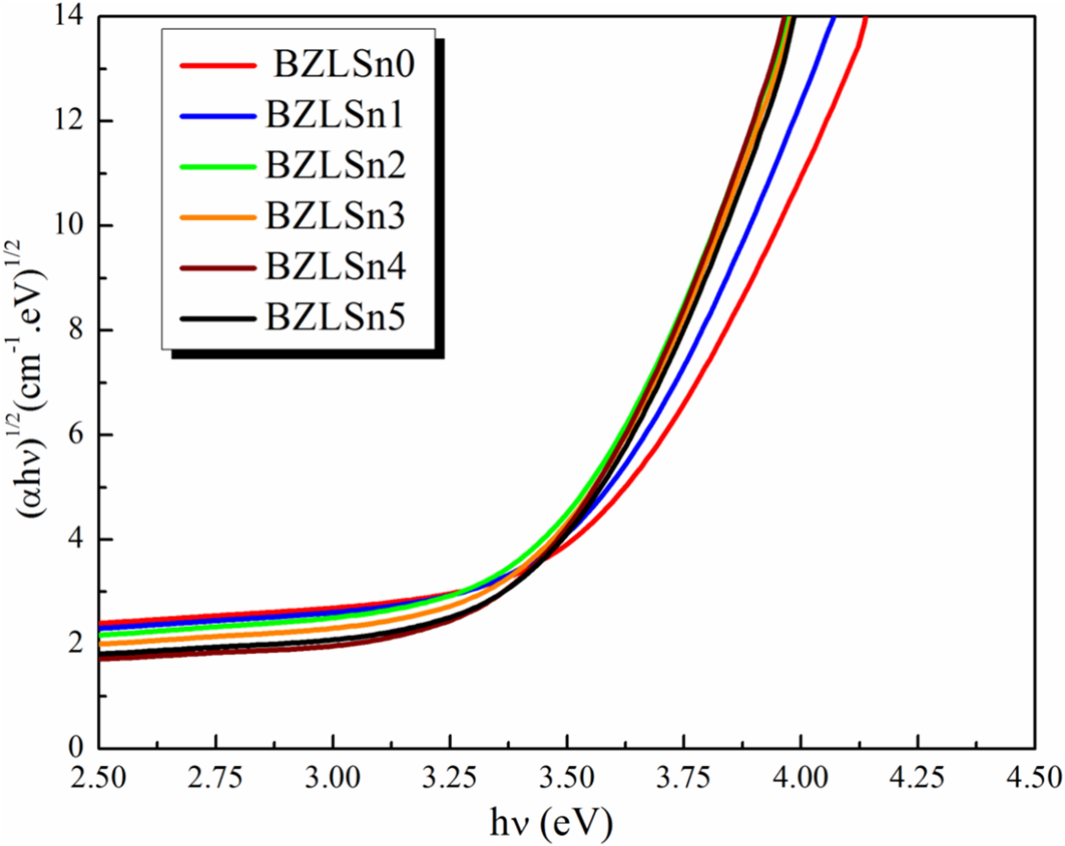
Dependence of (αhν)^1/2^ on (hν) for the indirect allowed transition of the examined BZLSn samples.

Figures 4 and 5 show the dependence of (αhν)^2^ and (αhν)^1/2^ on (hν) for the direct and indirect allowed transitions of the examined samples. Table 2 summarizes the E_g_^optical^ values of both the direct and indirect transitions of undoped and doped SnO_2_ glass samples. The E_g_^optical^ values decrease during the direct and indirect transitions when the SnO_2_ concentration in the produced glasses is increased.

**Table 2:** Optical parameters of the BZLSn glasses.

Sample	E_g_ (eV)		E_g_^Optical^	n	E_u_ (eV)
	Direct	Indirect			
BZLSn0	3.257	2.902	3.079	2.376	0.251
BZLSn1	3.230	2.855	3.043	2.386	0.234
BZLSn2	3.202	2.825	3.014	2.393	0.219
BZLSn3	3.201	2.820	3.011	2.394	0.214
BZLSn4	3.198	2.817	3.008	2.395	0.211
BZLSn5	3.197	2.815	3.006	2.395	0.208

The difference between the Eg of SnO_2_ (3.6 eV)^30^ and the large band gap of LiF (14 eV)^31^ may be related to the decreasing optical energy values E_g_ with increasing the content of SnO_2_.

Equation (4) is used to compute the refractive index values (n) of the manufactured samples during the direct and indirect allowed transitions^32^:

4 $$\left( {\frac{{n^{2} - 1}}{{n^{2} + 2}}} \right) = 1 - \sqrt {\frac{{E^{optical} }}{20}}$$

Table 2 lists the values of (n) for all synthesized samples. Clearly, the optical energy band gaps are inversely related to the refractive index (n) of all the manufactured glasses. The refractive index increases from 2.376 to 2.395, indicating that the proposed glasses may be used as a promising material for optical filters and photoelectronic applications.

Th absorption coefficient (α) can be estimated using Urbach's empirical formula^33,34^:

5 $$\alpha = \alpha_{0} exp\left( {\frac{hv}{{E_{u} }}} \right)$$

where $\alpha_{0}$ is a constant and $E_{U}$ is the Urbach energy. Equation (5) can be written as:

6 $$ln\alpha = ln\alpha_{0} + \left( {\frac{hv}{{E_{u} }}} \right)$$

**Figure 6:**
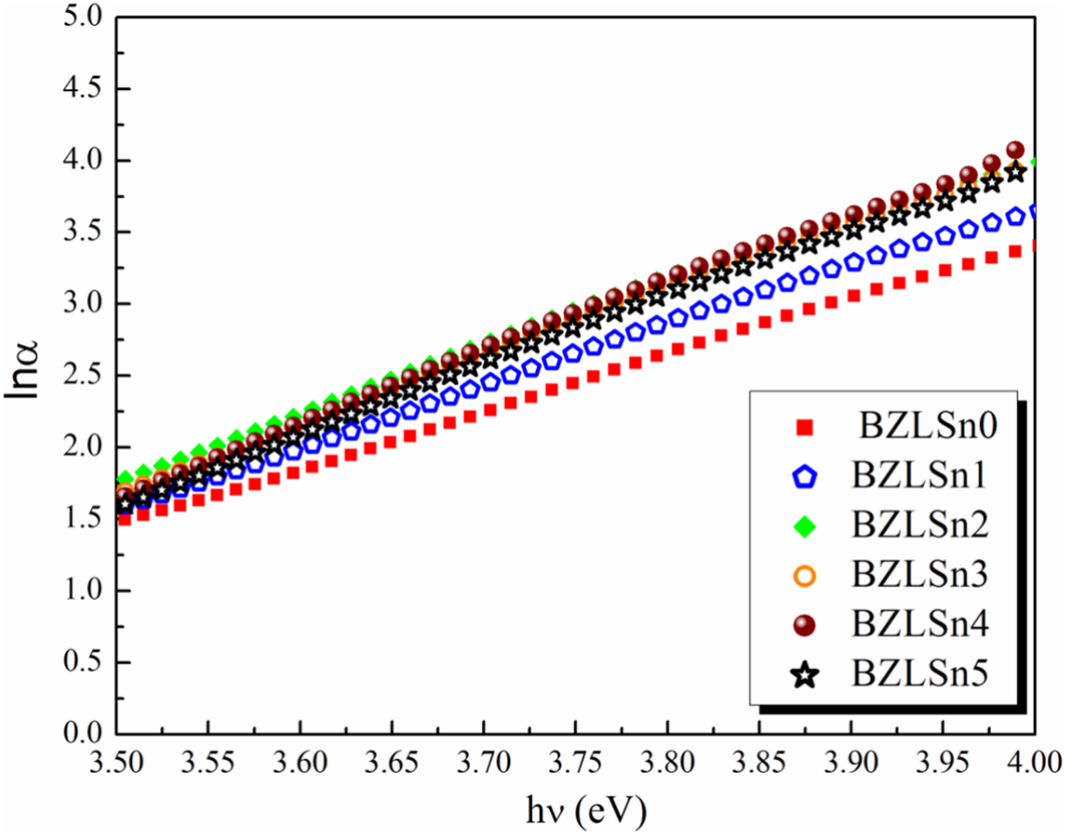
Change of ln(α) with respect to hν of the prepared BZLSn samples.

As a consequence, the slope of the straight line obtained by plotting ln(α) vs. (hv) may be used to calculate Urbach's energy (E_u_). The change in ${\text{ln}}\left( \alpha \right)$ with photon energy ($h\upsilon$) is exhibited in Fig. 6. As listed in Table 2, the Urbach energies decrease as the SnO_2_ concentration is increased, indicating an increase in stability and decrease in disorder in the glass samples.

### Mechanical properties of the fabricated samples

**Figure 7:**
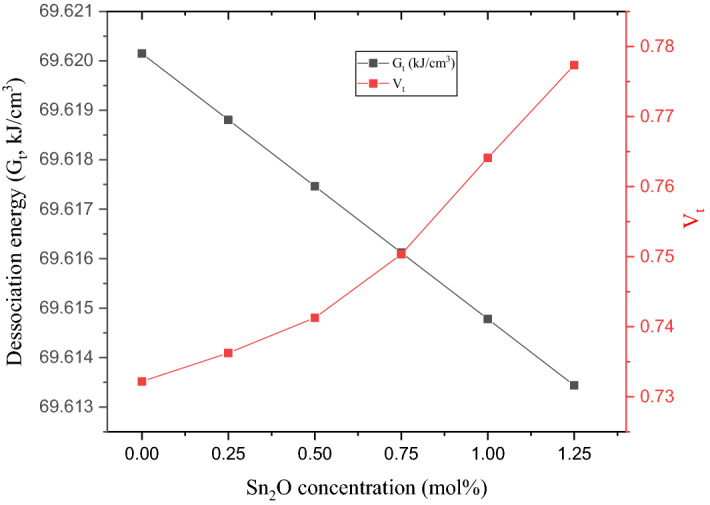
Dissociation energy (G_t_, kJ/cm^3^) and packing density (V_t_) versus the increasing Sn_2_O ratio.

The effect of replacing LiF with SnO_2_ on the dissociation energy (G_t_) and packing factor (V_t_) of the fabricated glasses was studied. Figure 7 depicts a linear decrease in G_t_ as well as an increase in V_t_ with an increasing replacement ratio of LiF by SnO_2_. The G_t_ values decrease slightly from 69.620 to 69.613 kJ/cm^3^, while the V_t_ values increase from 0.732 to 0.777 when the SnO_2_ insertion ratio is increased from 0.0 to 1.25 mol%, respectively. The mentioned decrease in the G_t_ values is due to the replacement of the LiF compounds with G_t_ = 38.801 kJ/cm^3^ by SnO_2_ with a comparable G_t_ value where (G_t_)_SnO2_ = 38.264 kJ/cm^3^. The increase observed in the Vt values is due to the replacement of a low packing factor (V_i_) compound with a compound having a higher one; for instance, the V_i_ of LiF is 4.894 cm^3^/mol while it is 13.862 cm^3^/mol for SnO_2_.

**Figure 8:**
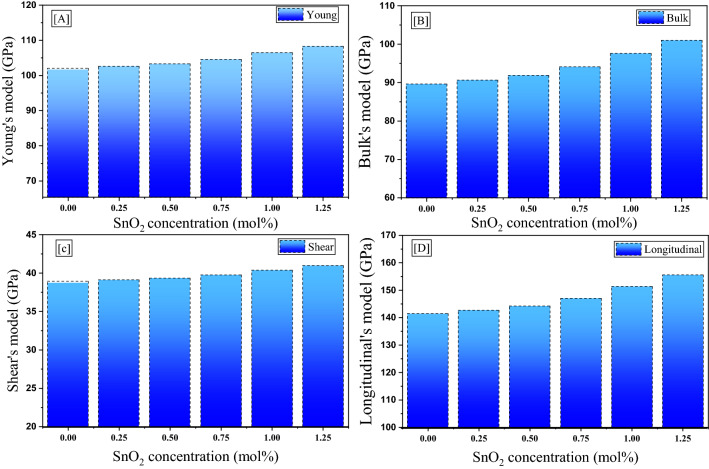
Effect of increasing SnO_2_ on the elastic moduli of the fabricated glass system.

Figure 8 shows the effect of replacing LiF with Sn_2_O on the elastic moduli of the fabricated glasses. It is known that the elastic moduli are related and proven from the predicted values of both G_t_ and V_t_ presented in the previous section. According to the results presented in Fig. 8 A, the Young modulus increases from 101.949 GPa to 108.226 GPa when the Sn2O insertion ratio is increased from 0.0 to 1.25 mol%, respectively. This increase in the Young modulus is directly related to the increase achieved for the V_t_ of the fabricated glasses when LiF is replaced by SnO_2_, where Y = 2V_t_G_t_. Additionally, Fig. 8B,C,D show that the bulk (B), shear (S), and longitudinal (L) moduli follow the same trend as the Young modulus. The bulk modulus increases from 89.574 to 100.953 GPa, the shear modulus (S) increases from 38.903 to 40.953, and the longitudinal modulus exhibits the greatest values, increasing from 141.444 to 147.030 GPa when the SnO_2_ ratio is increased from 0.0 to 1.25 mol%.

**Figure 9:**
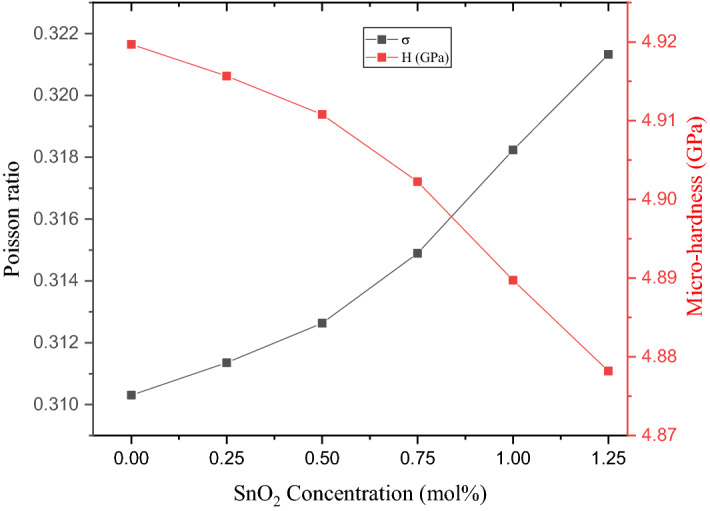
Poisson ratio and microhardness as a function of the Sb_2_O_3_ ratio.

The Poisson ratio (σ) is a mechanical parameter that measures the ratio of glass deformation in a direction perpendicular to the loading direction. According to the theoretical calculations, the Poisson ratio is based on the predicted values of the Young (Y) and Shear moduli, where $\sigma =\frac{E }{2 S}-1$. In the present work, as illustrated in Fig. 9, the σ values increase from 0.310 to 0.321 when the SnO_2_ ratio is increased from 0.0 and 1.25 mol%. The mentioned increase is related to the increase recorded in the V_t_ and shear moduli (S). The microhardness (H, GPa) is a factor used to describe the hardness of the fabricated glass samples at the microscale, and it is proof of the Poisson ratio (σ), where H = [(1-2σ)*V_t_]/[6(1 + σ)]. The H calculations show that the H values slightly decrease from 4.920 to 4.878 GPa when the SnO_2_ is increased from 0 to 1.25 mol% in the fabricated samples. This slight decrease is related the fabricated glasses’ Poisson ratio and V_t_.

### Gamma-ray shielding properties

**Figure 10:**
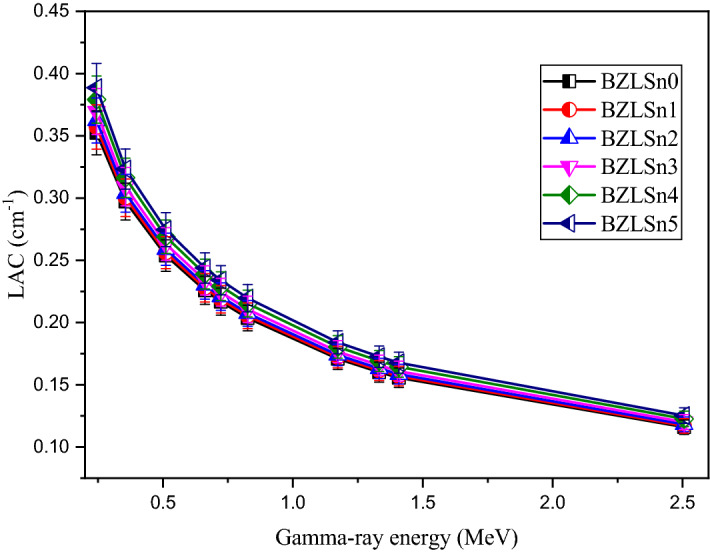
Changes in the linear attenuation coefficient (LAC, cm^−1^) as the gamma photon energy is increased from 0.2234 to 2.506 MeV.

The shielding capacity of SnO_2_-based zinc lithium borate glasses was evaluated using Monte Carlo simulation^35^ and the XCOM^36^ theoretical program between 0.2234 and 2.506 MeV. First, the obtained average track length (ATL) of the gamma photons was evaluated based on the MC simulation. Then, the ATL was transferred to the linear attenuation coefficient (LAC, cm^−1^) using Lambert–Beer's law. The MC evaluated results were verified using the XCOM program at the same energies as the MC simulation. Figure 10 illustrates that the LAC of the fabricated SnO_2_-based borate glasses suffers a moderately exponential decrease as the energy is increased from 0.2234 to 2.506 MeV. This exponential decrease is due to Compton scattering (CS), representing the primary interaction in the selected energy region. The cross-section corresponding to CS decreases linearly with increasing photon energy, where σ_Cs_ α E^−1^ ^37^. When the photon energy is varied from 0.244 to 2.506 MeV, the LAC decreases in the following intervals: 0.352–0.116, 0.357–0.117, 0.362–0.118, 0.369–0.120, 0.379–0.122, and 0.388–0.125 cm^−1^ for samples BZLSn0, BZLSn1, BZLSn2, BZLSn3, BZLSn4, and BZLSn5, respectively. The increase in the energy of the incident photon increases the frequency and penetration power of the incident photon. Thus, the photon cross-section of the interaction decreases. Therefore, the decreases in the interaction probability are associated with the significant decreases in the linear and mass attenuation values.

**Figure 11:**
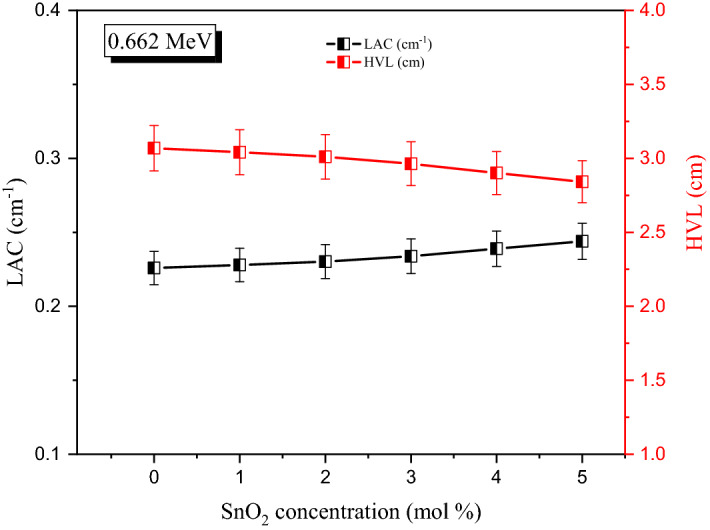
Linear attenuation coefficient (LAC, cm^−1^) and half-value layer (HVL, cm) with respect to the SnO_2_ concentration at 0.662 MeV.

The effect of the added SnO_2_ appears clearly in Fig. 11, where the LACs of the BZLSn glasses increase with increasing replacement of LiF by SnO_2_. At a gamma energy of approximately 0.662 MeV, the LAC increases in the order of 0.226, 0.228, 0.230, 0.234, 0.239, and 0.244 cm^−1^ when the amount of SnO_2_ added into the glass matrix is 0, 1, 2, 3, 4, and 5 mol%, respectively. This increase in the LACs is caused by the replacement of LiF (with a lower density and absorption cross-section (ρ_LiF_ = 2.64 g/cm^3^)) with SnO_2_ (with a higher density and photon absorption cross-section(ρ_SnO2_ = 6.95 g/cm^3^)). As a result of the replacement of a light compound (LiF) with a dense compound, the probability of the interaction between the incident photons and the distributed atoms and electrons increases due to the increase in the interaction cross-section. This increase in the interaction cross-section is reflected in the LACs and MACs, where both the LAC and MAC increase with increasing SnO_2_ ratio in the fabricated samples. The enhancement of the LACs and MACs is positively reflected in other shielding parameters, as illustrated in Fig. 11. The thickness required to decrease the source radioactivity to half (half-value layer, HVL) is enhanced by adding SnO_2_ into the glass matrix. The HVL regularly decreases with increasing LiF substituted by SnO_2_. Among the current study, thinner HVLs are achieved at 0.223 MeV. The HVL decreases from 1.967 to 1.784 cm when the SnO_2_ concentration is increased from 0 and 5 mol%. On the other hand, the thicker layers achieved at 2.506 MeV decrease in the following order: 5.975, 5.923, 5.863, 5.773, 5.652, and 5.535 cm for the prepared BZLSn0, BZLSn1, BZLSn2, BZLSn3, BZLSn4, and BZLSn5 samples, respectively. Additionally, Fig. 11 shows that, at a gamma-ray energy of 0.662 MeV, the HVL decreases from 3.069 to 2.845 cm as the SnO_2_ ratio is increased from 0 to 5 mol%. For all fabricated BZLS glasses, the HVL reduction is due to the LAC being enhanced with increasing SnO_2_ addition, where HVL = 0.693/LAC.

**Figure 12:**
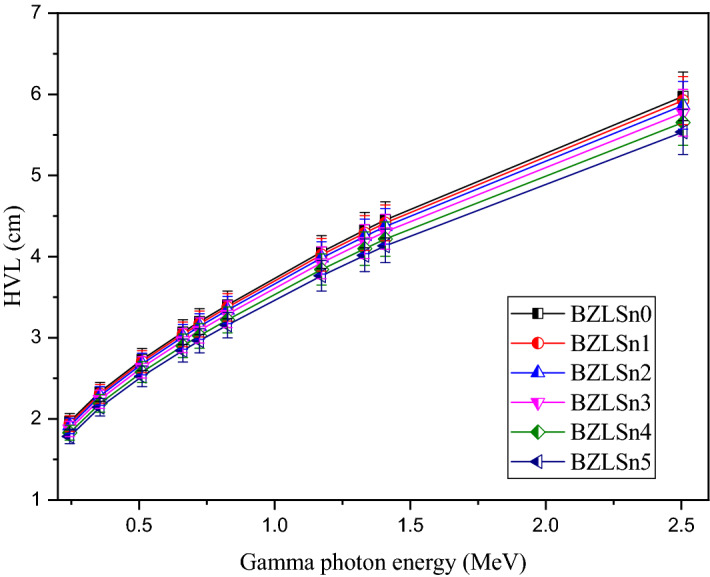
Half-value layer (HVL, cm) of the fabricated BZLSn glasses as a function of the gamma photon energy.

Figure 12 illustrates the gamma photon energy effects on the HVL values. Figure 12 shows an increase in the HVL associated with raising the incident gamma photon energy from 0.223 to 2.506 MeV. For all samples, the HVL linearly increases with the incident energy. For example, the HVL increases from 1.967 to 5.975 cm for BZLSn0, while it increases from 1.784 to 5.535 cm for BZLSn5 when the gamma-ray energy is increased from 0.223 to 2.506 MeV, respectively. The average HVL in the investigated energy range is approximately 3.551, 3.519, 3.483, 3.428, 3.355, and 3.286 cm for the BZLSn0, BZLSn1, BZLSn2, BZLSn3, BZLSn4, and BZLSn5 glasses, respectively. The increase in the HVL with energy is related to the penetration power of the incident gamma ray, where the gamma-ray penetration power increases with increasing energy. Thus, the transmission factor (Io/I) increases with the penetration power, and the thickness required to attenuate half of the incident photons will increase as a result.

**Figure 13:**
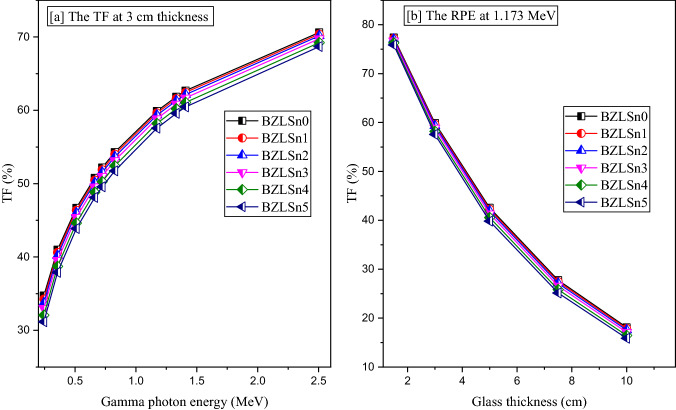
Relation between the transmission factor (TF, %) and the (**a**) gamma photon energy and (**b**) glass thickness.

The transmission factor is a measure of the number of photons that penetrate the shielding thickness after a collision or without collisions^38^. It is mainly dependent on three parameters: the source energy, chemical composition of the shielding material, and thickness. Figure 13a shows the dependence of the gamma photon transmission factor (TF) on the source energy, while Fig. 13a illustrates the effect of material thickness on the transmission ability of gamma photons. According to Fig. 13a, the TF regularly increases with increasing source energy. The lowest TFs are approximately 43.74, 34.36, 33.73, 33.00, 32.06, and 31.17% for BZLSn0, BZLSn1, BZLSn2, BZLSn3, BZLSn4, and BZLSn5, respectively. On the other hand, the highest TF is achieved at 2.506 MeV equaling 70.61, 70.39, 70.14, 69.75, 69.22, 68.68 for the previously mentioned glasses. As mentioned in the HVL part, the TF is related to the photon penetration power. Thus, the resistance and attenuation of the shielding materials decrease with increasing source energy, so the number of photons that can escape and penetrate the shielding material thickness increase as a result. In contrast, Fig. 13b shows a significant decrease in the TFs of all prepared glass samples with increasing glass thickness. Among the selected thicknesses, the highest TFs are from glass with a thickness of 1.5 cm. The TFs at 1.173 MeV vary, with values of 77.39, 77.22, 77.03, 76.72, 76.29, and 75.87% for glasses with SnO_2_ concentrations of 0, 1, 2, 3, 4, and 5 mol%, respectively. The lowest TF is achieved for materials with a thickness of 10 cm. The TFs vary between 18.11 and 15.86%. Increasing the glass thickness forces the incident photons to undergo more collisions before passing the glass layer. Thus, the attenuation of photons increases, and the TF decreases significantly. Additionally, the replacement of light compounds, such as LiF, with a denser compound, such as SnO_2_, increases the net density of the glass, which affects the attenuation of photons inside the shielding layers. Therefore, the replacement of LiF with SnO_2_ reduces the transmission of photons through the BZLSn glasses. The small variation in the TFs among the glass samples is related to the small amounts of LiF displaced by SnO_2_, where the highest SnO_2_ concentration is only 5 mol%.

**Figure 14:**
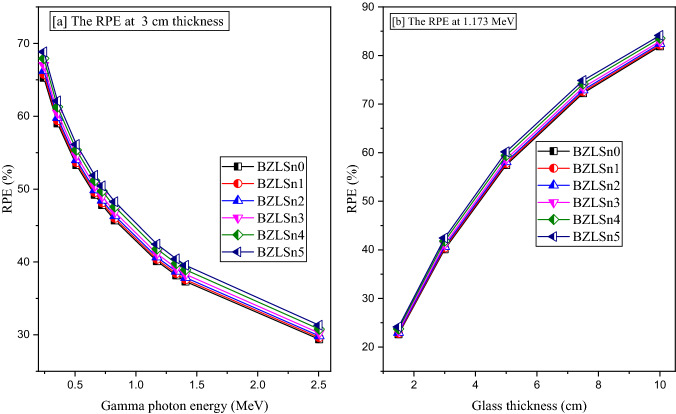
Variation in the radiation protection efficiency (RPE, %) versus the gamma photon energy and glass thickness.

The radiation protection efficiency (RPE, %) is used to describe the energy deposited inside the fabricated BZLSn glasses. Figures 14a and b depict the RPE variation with respect to the incident gamma photon energy and glass thickness. In the case of Fig. 14a, the RPE shows a linearly decreasing trend with increasing energy from 0.244 to 2.506 MeV. Among the studied energies, the best RPE is recorded at 0.244 MeV. Notably, the RPE varies, with values of 65.261, 65.743, 66.999, 67.936, 68.833% when the content of SnO_2_ is 0, 0.25, 0.5, 0.75, 1.0, and 1.25 mol%, respectively (representing BZLSn0, BZLSn1, BZLSn2, BZLSn3, BZLSn4, and BZLSn5). These results indicate that at a gamma-ray energy of 0.244 MeV, the photons undergo many collisions with the glass atoms and lose a very large amount of their energy inside the glass layer. Thus, only a small number of photons can penetrate the glass and reach exposed workers. Then, with increasing gamma photon energy, the photon penetration power increases, the number of photon collisions decreases, and the energy transferred from photons to the glass layer decreases with increasing number of photons that can penetrate the glass layer. Hence, the number of photons reaching workers increases with a decrease in the glass thickness (i.e., RPE). Among the studied energies, the lowest RPE is recorded at 2.506 MeV, and the values vary in the order of 29.390, 29.607, 29.858, 30.248, 30.780, and 31.317% for BZLSn0, BZLSn1, BZLSn2, BZLSn3, BZLSn4, and BZLSn5, respectively.

The glass thickness also greatly affects the protection capacity of the shielding material. As illustrated in Fig. 14b, the RPE increases linearly with increasing BZLSn glass thickness from 1.5 to 10 cm. Among the studied glass thicknesses, the lowest RPE is obtained at a thickness of 1.5 cm from the fabricated BZLSn glass samples. At 1.173 MeV, the recorded RPE for a thickness of 1.5 cm is 22.609, 22.777, 22.973, 23.281, 23.713, and 24.133 cm. In addition, the highest level of protection is obtained from the thicker samples with a thickness of 10 cm, where the RPE varies, with values of 81.889, 82.149, 82.449, 82.912, 83.544, and 84.139% for samples BZLSn0, BZLSn1, BZLSn2, BZLSn3, BZLSn4, and BZLSn5, respectively. It is clear that increasing the glass thickness causes an increase in the path length of the gamma photons inside the investigated samples, which forces the incident photons to undergo additional collisions with glass atoms and electrons. Thus, the energy deposited inside the glass layer increases, and the number of photons that can escape from the glass thickness decreases. Therefore, the RPE ratio increases.

**Figure 15:**
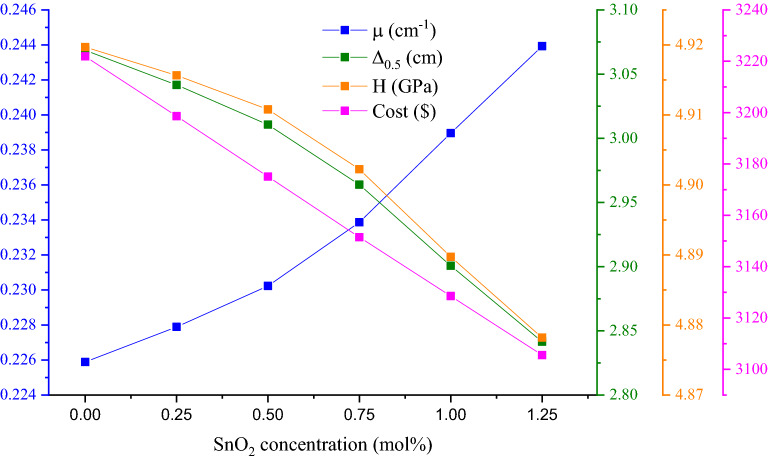
Variation of the linear attenuation coefficient, half-value layer, manufacturing cost, and microhardness versus the SnO_2_ insertion ratio.

To select the best fabricated glass that possesses suitable shielding and mechanical properties, as well as being inexpensive to fabricate, the variations of the LAC, HVL, microhardness, and fabrication cost versus the SnO_2_ concentrations were studied; the results are shown in Fig. 15. As mentioned in the last paragraphs, the insertion of SnO_2_ causes a significant increase in the µ values due to the significant increase in the packing factor V_t_ and bulk density associated with the replacement of LiF by SnO_2_. The significant increase in the packing factor causes an increase in the Poisson ratio associated with a decrease in the microhardness of the material. According to the mentioned figure, the best sample in the present study is has a SnO_2_ concentration of 0.75 mol% (i.e., BZLSn3). The µ value of the mentioned sample is 0.2338 cm^−1^, and the HVL is 2.9638 cm at 0.662 MeV. Additionally, the mentioned sample contains a microhardness value of 4.9022 GPa. Furthermore, the fabrication cost for a sheet of BZLSn3 glass with dimensions of 100 cm × 100 cm, which can attenuate half of the incident photons when the energy is 0.662 MeV, is approximately $3151.46. This relatively high cost is due to LiF being expensive, which costs $0.384/g of fabricated sheet. Therefore, the replacement of LiF by SnO_2_ causes a significant decrease in the fabrication cost due to SnO_2_ having a relatively low price compared to LiF (SnO_2_ costs approximately $0.05/g).

## Conclusion

In the present study, the effect of increasing SnO_2_ in a glass system based on zinc lithium borate was studied. The fabricated glass density increased from 3.025 and 3.265 g/cm^3^, while the molar volume decreased from 21.628 to 20.516 cm^3^/mol for the fabricated BZLSn glasses when the content of SnO_2_ was increased from 0 to 1.25 mol%. According to the optical property calculations, the energy gap during direct and indirect transitions decreased from 3.257 to 3.197 eV and from 2.902 to 2.815 eV, respectively. The Monte Carlo simulation showed that BZLSn5 with a SnO2 composition of 1.25 mol% had the highest shielding capacity among the fabricated samples. The linear attenuation coefficient of the previously mentioned sample decreased from 0.389 to 0.125 cm^−1^, and the half-value layer increased from 1.784 to 5.535 cm as the photon energy was increased from 0.244 to 2.506 MeV. The previously illustrated results showed that the replacement of LiF by 1.25 mol% SnO_2_ resulted in an enhanced shielding capacity, reaching 9.3% at 0.244 MeV.
